# Natural history of valve disease in patients with mucopolysaccharidosis II and the impact of enzyme replacement therapy

**DOI:** 10.1002/jimd.12808

**Published:** 2024-10-23

**Authors:** Christoph Kampmann, Christina Lampe, Christiane M. Wiethoff, Laila Arash‐Kaps, Eugen Mengel, Joerg Reinke, Michael Beck, Julia B. Hennermann, Tariq Abu‐Tair

**Affiliations:** ^1^ Department of Pediatric Cardiology and Structural Heart Diseases, Center for Diseases in Childhood and Adolescence University Medicine Mainz Mainz Germany; ^2^ Department of Child Neurology, Epileptology and Social Medicine University Hospital Giessen Giessen Germany; ^3^ SphinCS, Institute of Clinical Science in LSD Hochheim Germany; ^4^ Medical center for adults with disabilities Kreuznacher Diakonie Bad Kreuznach Germany; ^5^ Department of Metabolic Diseases, Villa Metabolica, Center for Diseases in Childhood and Adolescence University Medicine Mainz Mainz Germany

**Keywords:** glycosaminoglycans, heart valve disease, Hunter syndrome, lysosomal storage disorder

## Abstract

Mucopolysaccharidosis II (MPS II, Hunter syndrome) is a rare, X‐linked lysosomal storage disease caused by reduced activity of iduronate‐2‐sulfatase (I2S), with subsequent cellular accumulation of the glycosaminoglycans (GAGs), heparan sulfate, and dermatan sulfate (DS). DS is a major component of the extracellular matrix of heart valves, which can be affected in MPS II. We investigated the natural history of valve disease in MPS II and the impact of long‐term intravenous enzyme replacement therapy (ERT) with recombinant I2S (idursulfase). In total, 604 cardiac examinations were assessed from serial follow‐up of 80 male patients (49 neuronopathic). Valve disease was classified according to standard practice from hemodynamic features evident from echocardiography. The natural history group comprised 48 patients (up to 14.8 years of follow‐up; median, 2.6 years; 24 patients started ERT during the study); 56 patients were treated (up to 14.2 years of follow‐up; median, 6.2 years). Lifetime GAG burden (calculated from urinary GAG measurements) correlated significantly with the degree of valve disease. Onset of moderate‐to‐severe valve disease was significantly delayed in treated (median age at onset, 29.1 ± 2 [95% CI: 25.2–32.9] years; Kaplan–Meier estimation) versus untreated patients (17.6 ± 1 [95% Cl: 15.8–19.4] years; *p* < 0.0001). Cox regression modeling found that long‐term ERT reduced the probability of developing severe valve disease (*χ* ^2^, 32.736; significant after 5 years of ERT). Overall, this study found that valve disease severity in MPS II correlates with GAG burden and that progression is delayed by long‐term ERT.

## 1 INTRODUCTION

Mucopolysaccharidosis II (MPS II, Hunter syndrome; OMIM 309900), is a rare X‐linked, progressive lysosomal storage disease, characterized by deficiency in the activity of the enzyme iduronate‐2‐sulfatase (I2S),^1^, ^2^ which results in accumulation of the glycosaminoglycans (GAGs), heparan sulfate (HS), and dermatan sulfate (DS). MPS II occurs nearly exclusively in males, with an estimated birth prevalence of 0.13–2.16 per 100 000 live births,^3^ and is a heterogeneous disorder with regard to age at symptom onset, severity, and rate of progression.^4^ An estimated two‐thirds of patients have the more severe neuronopathic form,^5^ experiencing progressive cognitive impairment and developmental regression in addition to somatic involvement, and typically dying in the second decade of life.^1^, ^4^ Patients with an attenuated, non‐neuronopathic phenotype may have normal cognitive ability and often survive into adulthood, but still experience somatic manifestations of the disease. Data on genotype–phenotype correlations in MPS II are limited, although patients with large deletions or rearrangements in the *IDS* gene resulting in complete loss of functional enzyme have the neuronopathic form of MPS II as well as severe somatic manifestations, including cardiac involvement.^6^, ^7^, ^8^, ^9^ Due to the multi‐systemic and progressive nature of the disease, patients with MPS II require multi‐disciplinary care and typically undergo multiple surgeries.

Cardiac involvement contributes substantially to morbidity and mortality in MPS II: half of patients exhibit cardiovascular involvement by 8 years of age, rising to 70% by 15 years of age.^2^, ^9^, ^10^, ^11^, ^12^, ^13^ Such involvement in patients with MPS II includes valve disease, cardiomyopathy, arrhythmias and abnormal tachycardia, congestive heart failure, atrial and ventricular volume overload, diastolic dysfunction, and, rarely, endocardial fibroelastosis. Valve disease is the most common cardiac manifestation, reported to occur in over 60% of untreated patients and with a median age of onset of 5.8 years.^9^

DS is a major component of the extracellular matrix of the heart and heart valves,^14^, ^15^ and accumulation of DS products leads to significant alterations in the aortic and mitral valves in patients with MPS II.^10^ This most commonly manifests as valve regurgitation, stenosis, or a combination of both. In severe cases of valve disease, valve replacement is needed. Intravenous enzyme replacement therapy (ERT) with idursulfase (Elaprase®; Shire [a Takeda company], Lexington, Massachusetts, USA) became available for the treatment of somatic aspects of MPS II in 2006 and has been shown to prevent or stabilize left ventricular hypertrophy (LVH), but only limited data are available on the influence of treatment on valve thickening or valve disease.^11^, ^16^, ^17^, ^18^ In addition, there have been few studies on the natural history of valve disease, and the majority are cross‐sectional analyses; there remains a need for an in‐depth longitudinal analysis of the severity and progression of valve disease in MPS II.

The purpose of this single‐center, longitudinal study was to describe the natural history of valve disease in the long‐term follow‐up of a large cohort of untreated patients with MPS II, and to examine the impact of long‐term ERT.

## 2 METHODS

### 2.1 Patients

Patients with MPS II have been followed at the metabolic and the cardiac units at the University Children's Hospital in Mainz, Germany, since 1993. Over the years, 81 patients with MPS II have been evaluated (all male). Included within the cohort were 12 siblings from six families; in these families, diagnosis of the younger brother was achieved at an early age based on family history. In one family with two sons, MPS II was diagnosed in the younger brother at 6 weeks of age. Because the older brother had the neuronopathic phenotype, the younger brother received intravenous ERT for 6 months, at which point hematopoietic stem cell transplantation was performed. This patient was excluded from this study, resulting in a total of 80 patients. Patients who were followed before ERT became available or whose parents and guardians denied ERT served as a natural history (untreated) group. Forty‐eight of the 80 patients had a period of being untreated during this study; of these 48, 24 started ERT during the study and 24 remained untreated throughout. Thirty‐two patients received ERT for the full study duration. Assessment of neuronopathic or non‐neuronopathic phenotype was by clinical impression based on the experience of the managing physician.

### 2.2 Cardiac examinations and grading of valvular disease

On average, 3.4 cardiac examinations were available per patient in the natural history group (maximum, 16) and 8.6 in the treated group (maximum, 32). A total of 604 cardiac examinations could be matched to patient age for analysis of valve disease in treated and untreated patients: 190 examinations in 48 patients in the natural history group and 414 examinations in 56 patients receiving ERT. Echocardiograms were not available for one patient. All 604 echocardiograms were blinded and were analyzed retrospectively by a single cardiologist. Alterations observed in the valves were assessed according to recommendations from the American Society of Echocardiography.^19^, ^20^, ^21^ Valve alterations were classified into the following three groups: thickening of valvular leaflets without any stenosis or regurgitation, degree of regurgitation, and degree of stenosis. The degree of regurgitation was recorded and classified as grade 0 (no regurgitation), grade 1 (minimal), grade 2 (minor to moderate), grade 3 (moderate) and grade 4 (severe regurgitation) for every valve. The degree of stenosis of atrioventricular valves was classified as grade 0 (normal inflow), grade 1 (accelerated inflow with a gradient below or equal to 2.5 mmHg in mean), grade 2 (above 2.5 but below or equal to 5 mmHg in mean), grade 3 (mean gradient above 5 mmHg but below or equal to 10 mmHg) and grade 4 (above 10 mmHg in mean), and for the semilunar valves in grade 1 (peak gradient below 20 mmHg) grade 2 (peak gradient below 50 mmHg) and grade 3 all above 50 mmHg.

After grading the different valve classifications, we developed a second grading to describe the hemodynamic consequences of the valve disease and thereby simplify the overall classification. Grade 0 (no valve disease); grade 1 (minimal valve disease, comparable to grade 1 of the isolated valve classifications); grade 2 (mild valve disease, without ventricular or atrial compensation, hypertrophy or enlargement, comparable to grade 2 of the isolated valve classifications); grade 3 (moderate but significant valve disease, comparable to grade 3 of the former classifications, except for the classifications of semilunar valve stenosis, which are comparable to grade 2, and comprising left atrial enlargement of >35 mL/m^2^, left ventricular hypertrophy of >115 g/m^2^ in male patients and >95 g/m^2^ in female patients and left ventricular dilation of >75 mL/m^2^ in male patients, and >61 mL/m^2^ in female patients according to recommended thresholds^22^); and grade 4 (severe valve disease, comparable to the highest grades of the isolated valve classifications, and comprising severe left atrial enlargement of >42 mL/m^2^, severe left ventricular hypertrophy of >115 g/m^2^ in male patients and >95 g/m^2^ in female patients and left ventricular dilation of >75 mL/m^2^ in male patients and >61 mL/m^2^ in female patients according to recommended thresholds^22^). Patients who underwent valve surgery remained classified as grade 4 (severe valve disease). This revised grading system (grades 0–4) was then used to classify severity of valve disease; the corresponding descriptions of “mild,” “moderate,” or “severe” are used in the reported results to aid interpretation.

### 2.3 Estimate of lifetime GAG burden

Hypothetically, in chronic progressive metabolic diseases, the exposure to an undegraded metabolic product and the result of organ or cellular dysfunction follows a function of time. Therefore, measurements of excreted GAGs (indirectly reflecting DS levels) were obtained using an assay based on 1,9‐dimethylmethylene blue binding, as described previously,^23^ and first normalized to excreted creatinine (crea) from 24‐hour urine samples. To calculate the lifetime GAG burden, the GAG/creatinine ratio from the earliest assessment was multiplied by the age of the patient in days (D) at the time that the measurement was performed, and any additional GAG/creatinine measurements were multiplied by the number of days since the previous assessment before adding to the total. This method offers an estimate of the lifetime amount of excreted GAGs, which roughly reflects the lifetime burden of the storage material in the individual patient.

### 2.4 Statistical analyses

Data are presented as mean values ± standard deviation (SD) with the 95% confidence limits (CLs) unless otherwise stated. *p* values were generated using an independent Student's t‐test for normally distributed data (assuming equal variances) or Wilcoxon rank‐sum test for data with a non‐normal distribution (assuming unequal variances); the significance threshold was set at a *p*‐value of <0.05. A Mantel–Cox regression model with *χ* ^2^ testing was used to compare between groups of valve disease. GAG data were presented using box plots. Differences in lifetime GAG burden between groups classified according to valve disease severity were investigated using Pearson's *χ* ^2^ test. Kaplan–Meier estimation was used to describe the onset of valve thickening and valve disease in relation to age of the patients depending on the status of ERT (Mantel–Cox log‐rank test). A Cox regression model adjusted for age at the start of ERT was used to discriminate between natural history follow‐up and follow‐up while patients were receiving ERT. No statistical correction was done on repeated testing.

## 3 RESULTS

### 3.1 Patient demographics and baseline cardiovascular characteristics

Overall, mean age at first presentation for the 80 patients in this study was 10.7 years (range, 1.8 months to 41.3 years). The majority of patients were of Western European ancestry (70 patients; 87.5%), seven patients (8.7%) were of Turkish origin and three (3.8%) were of Russian and Ukrainian origin. In total, six patients (three neuronopathic) died during this study: three (all non‐neuronopathic) from respiratory failure with cardio‐respiratory decompensation, two (both neuronopathic) from untreatable epileptic state and one (neuronopathic) from severe cardio‐embolic stroke. Survival status was unknown for seven patients.

Forty‐nine patients (61.3%) were classified as having neuronopathic disease and 31 (38.7%) had non‐neuronopathic disease. Baseline biometric and cardiovascular characteristics of all 80 patients in this study are shown in Table 1, presented according to neuronopathic or non‐neuronopathic phenotype. The non‐neuronopathic group was older at first cardiac examination than the neuronopathic group (*p* = 0.0001).

**TABLE 1:** Baseline biometric and cardiovascular data in patients in this study by the overall MPS II phenotype.

	Neuronopathic	Non‐neuronopathic	*p* value
Number of patients, *N*	49	31	
Baseline biometric data			
Age at first cardiac examination (years)	6.7 ± 4.4; 5.5 (0.15–21.2)	17.4 ± 11.5; 15.9 (1.85–41.3)	0.0001
Age at ERT start (years)^a^	7.9 ± 4.9; 6.1 (1.9–21.2)	19.7 ± 10.5; 19.3 (1.85–44.95)	0.0001
Weight (kg)	25.65 ± 9.9; 23.5 (4.3–53.3)	42.4 ± 19.8; 40.5 (12–93)	0.0001
Height (m)	1.1 ± 0.14; 1.14 (0.6–1.37)	1.33 ± 0.23; 1.36 (0.8–1.76)	0.0001
BSA (m^2^)	0.85 ± 0.22; 0.84 (0.24–1.28)	1.2 ± 0.38; 1.2 (0.53–1.93)	0.0001
BMI (kg/m^2^)	20.17 ± 5.4; 19.7 (9–40.3)	22.9 ± 6.7; 23.1 (11.7–44.6)	0.067
Baseline cardiovascular data			
Systolic blood pressure (mmHg)	112.7 ± 14; 112 (78–137)	120 ± 16; 115 (95–157)	0.085
Diastolic blood pressure (mmHg)	69.7 ± 10; 69 (51–100)	68.8 ± 12; 66 (46–95)	0.78
Presence of arterial hypertension, *n/N* (%)^b^	2/49 (4%)	1/31 (3.2%)	0.9^b^^,^ ^c^
Heart rate (bpm)^b^	101.9 ± 22.6; 96 (62–141)	86.8 ± 16.4; 89 (57–120)	0.003
Age‐related inappropriately high heart rate, *n/N* (%)	4/49 (8.2%)	0/31 (0%)	0.04
LV mass (g)	76 ± 32; 70 (15–184)	138 ± 73; 117 (42–288)	0.0001
LV mass indexed to BSA (LVMI; g/m^2^)	88 ± 24; 86 (45–150)	112 ± 47; 97 (53–244)	0.005
Presence of LVH, n/N (%) (LVMI ≥110 g/m^2^)	7/49 (14.3%)	14 / 31 (45.2%)	0.004^c^
EF (%)	63.5 ± 7.1; 62 (46–80)	66 ± 7.6; 67 (53–88)	0.158
Reduced systolic function (EF < 50%), *n/N* (%)	2/49 (4.1%)	1/31 (3.2%)	0.9^b^ ^,^ ^c^

*Note*: Data are presented as mean ± SD; median (range) unless otherwise stated. *p* values were generated using an independent Student's *t*‐test unless otherwise stated.

Abbreviations: BMI, body mass index; bpm, beats per minute; BSA, body surface area; EF, ejection fraction; ERT, enzyme replacement therapy; LV, left ventricular; LVH, left ventricular hypertrophy; LVMI, left ventricular mass index; MPS II, mucopolysaccharidosis II; SD, standard deviation.

^a^ Of the neuronopathic patients, 41/49 received ERT (one of these patients stopped ERT during the study); 25/31 non‐neuronopathic patients received ERT (one of these patients stopped ERT during the study).

^b^ Patients received 24‐hour blood pressure monitoring. Blood pressure and heart rate measurements were obtained at the time of echocardiogram assessment. Arterial hypertension was defined as a mean arterial blood pressure of > = 140/90 mmHg.

^c^ Wilcoxon rank‐sum test.

In the natural history (untreated) group (*n* = 48), median (range) follow‐up was 2.6 (0–14.8) years; five patients were followed for more than 12 years. In the treated group (*n* = 56; 24 patients initiated ERT after study start), ERT duration ranged from 1 to 14.2 years with a median of 6.2 years. Overall, median age at ERT start was 11.1 years (range, 1.85–44.95 years). Median age at ERT start was significantly lower (*p* = 0.0001) in those with the neuronopathic presentation (6.1 years; range, 1.9–21.2 years) than in those with the non‐neuronopathic form (19.3; 1.85–44.95 years); the proportion of the two phenotypes was similar in the treated and untreated groups.

### 3.2 Occurrence and type of valve disease

Valve disease was common, and present in almost half of patients with neuronopathic disease and two‐thirds of patients with non‐neuronopathic disease (Table 2). Valve thickening without stenosis or regurgitation (a pre‐cursor to valve disease) was observed in a high proportion of both neuronopathic and non‐neuronopathic patients.

**TABLE 2:** Valve disease in patients in this study by the overall MPS II phenotype.

	Neuronopathic		Non‐neuronopathic		*p* value^a^
	Stenosis	Re‐gurgitation	Stenosis	Re‐gurgitation	
*n/N*	10/49	14/49	15/31	15/31	0.07
Patients with valve disease, *n/N* (%)	20/49 (40.8%)		21/31 (67.7%)		0.06
Valve thickening without stenosis or regurgitation, *n/N* (%)	41/49 (83.7%)		30/31 (96.8%)		0.1
No valve disease, *n/N* (%)	29/49 (59.2%)		10/31 (32.3%)		
Mild valve disease, *n/N* (%)	11/49 (22.4%)		9/31 (29.0%)		
Moderate valve disease, *n/N* (%)	7/49 (14.3%)		8/31 (25.8%)		
Severe valve disease, *n/N* (%)	2/49 (4.1%)		4/31 (12.9%)		

Abbreviation: MPS II, mucopolysaccharidosis II.

^a^ Wilcoxon rank‐sum test.

Overall, the left‐side valves (mitral and aortic) were the most frequently affected. As expected for MPS II, aortic and mitral valve regurgitation were the most frequently occurring types of valve disease, with aortic and mitral valve stenosis also being common (Figure A). There were a small number of instances of pulmonary and tricuspid valve regurgitation, but the right‐side valves were otherwise unaffected.

Four patients underwent valve replacement surgery: two due to endocarditis and two due to progressive severe valve disease (all non‐neuronopathic phenotype, and receiving ERT). These patients were not excluded from further analyses because even endocarditis was considered a complication of valve disease.

### 3.3 Lifetime GAG burden, LVH, and valve disease

Overall, 201 GAG measurements could be correlated with the corresponding echocardiograms. Mean estimated lifetime GAG burden was significantly higher in patients with valve disease than in those without (565 485 ± 359 517 and 199 831 ± 92 977 (μg/mg_Crea_/d)*D, respectively; *p* = 0.0001) (Table A). Conversely, estimated lifetime GAG burden was similar in patients with and without LVH (388 091 ± 97 517 (μg/mg_Crea_/d)*D and 356 475 ± 328 952 (μg/mg_Cre_a/d) × D, respectively; *p* = 0.803). Left ventricular mass index (LVMI) was higher in patients with valve disease than in those without valve disease (*p* = 0.005).

**FIGURE 1:**
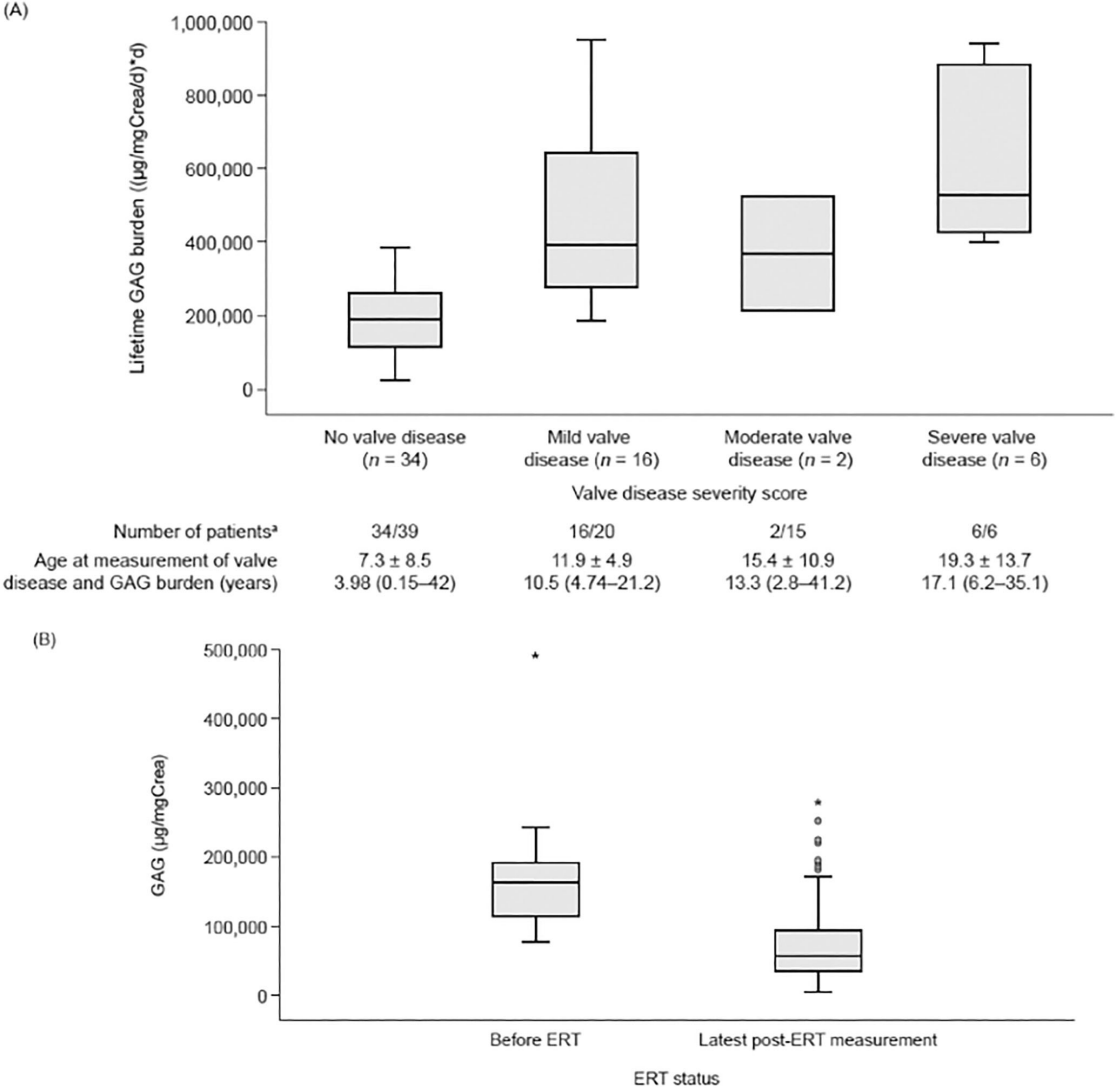
Glycosaminoglycan burden and association with valve disease severity and ERT in MPS II. (A) Severity of valve disease reflects the lifetime GAG burden. Box plot of most recent valve disease severity score against lifetime GAG burden, in untreated patients or before the start of ERT. *p* = 0.0001 for an association between increased severity of valve disease and increased lifetime GAG burden (Pearson's *χ* ^2^ test). (B) Box plot of urinary GAG levels before and after ERT. Measurements before and after ERT were available for 21 patients; the most recent post‐ERT measurement was used. GAG levels decreased by a mean ± SD of 83.4 ± 64.5 (95% CLs: −92, −74; *p* < 0.001) μg/mg_Crea_. Data are presented as mean ± SD; median (range) unless stated otherwise. CL, confidence interval; Crea, creatinine; ERT, enzyme replacement therapy; GAG, glycosaminoglycan; SD, standard deviation. The values marked with (*) are maximum values. Data were only included from patients with measurements available for both GAGs (before the start of ERT for treated patients) and valve disease.

In the natural history group, severity of valve disease increased with increased lifetime GAG burden (Figure 1A). When overall MPS II disease phenotype was considered, there was no difference in lifetime GAG burden between neuronopathic and non‐neuronopathic patients with a similar degree of valve disease (Table B). However, in both the neuronopathic and non‐neuronopathic groups, lifetime GAG burden was significantly higher in patients with moderate‐to‐severe valve disease than in those without valve disease (neuronopathic: 537950 ± 396 513 and 209 194 ± 94 149(μg/mgCrea/d) × D, respectively; non‐neuronopathic: 577722 ± 353 303 and 145 518 ± 70 335 (μg/mgCrea/d) × D, respectively; *p* < 0.01 for each).

Notably, GAG levels were significantly reduced with ERT (Figure 1B), by a mean ± SD of 83.4 ± 64.5 μg/mg creatinine (95% CLs, −92 to −74; *p* < 0.001).

### 3.4 Kaplan–Meier analyses of the impact of ERT on valve disease in MPS II

**FIGURE 2:**
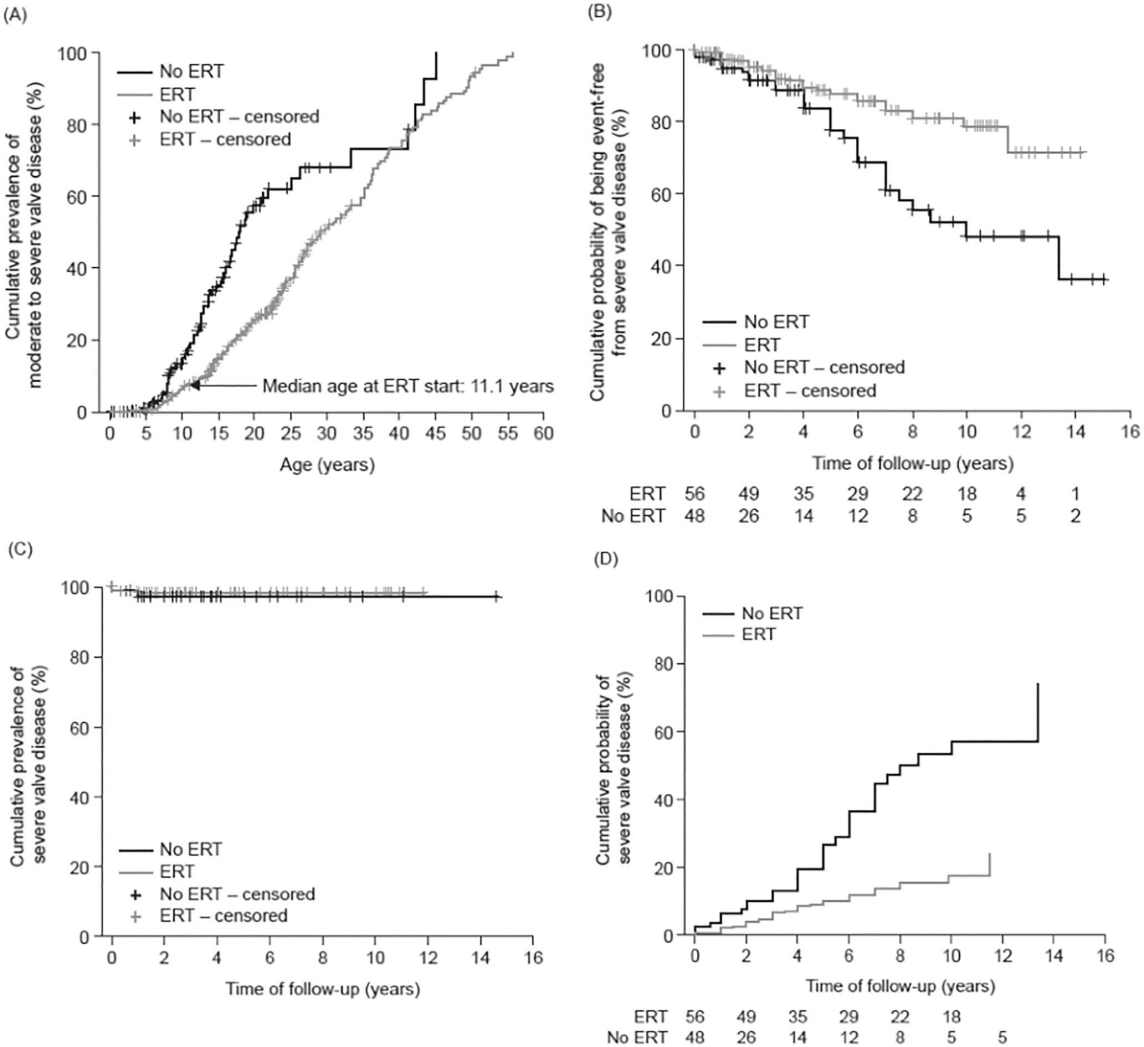
Impact of ERT on the valve disease. (A) Cumulative prevalence of moderate‐to‐severe valve disease in relation to age in untreated and enzyme‐treated patients, (B) Cumulative time event‐free from severe valve disease in treated and untreated patients, (C) Cumulative time event‐free from severe valve disease in non‐neuronopathic patients, (D) Cox regression model corrected for the means of covariant (mean age of ERT start): Cumulative probability for development of moderate‐to‐severe valve disease during follow‐up. (A) Median ± SE age at onset of moderate‐to‐severe valve disease in untreated patients: 17.6 ± 1 (95% CLs: 15.8–19.4) years; median age in treated patients: 29.1 ± 2 (95% CLs: 25.2–32.9) years; *χ* ^2^, 25.121; *p* < 0.0001; Mantel–Cox log‐rank test. Patients were censored at development of moderate‐to‐severe valve disease. (B) Median ± SE cumulative time event‐free from severe valve disease, 9.91 ± 0.69 (95% CLs: 8.56–11.3) years in untreated patients and not reached in treated patients; *χ* ^2^, 13.071; *p* < 0.0001; Mantel–Cox log‐rank test. Patients were censored at development of severe valve disease. (C) Median cumulative event‐free from severe valve disease remained unchanged in both treated and untreated non‐neuronopathic patients. (D) Probability of developing severe valve disease is reduced with ERT. *χ* ^2^, 32.736; *p* < 0.0001; Mantel–Cox omnibus test for coefficients. CI, confidence interval; ERT: Enzyme replacement therapy; N, number of patients; NS, not significant; SE, standard error. Data were from 56 treated patients (414 examinations) and 48 untreated patients (190 examinations). Mean follow‐up was 6.5 ± 3.9 years (median, 6.2 [range, 1.0–14.2] years) in treated patients and 4.3 ± 4.3 years (median, 2.6 [range, 0–14.8] years) (*p* = 0.014) in untreated patients. Mean age at first examination was similar in the treated and untreated groups: 13.5 ± 9.4 years (median, 11.8 [range, 2.9–45.2] years) and 9.6 ± 8.7 years (median, 7.1 [range, 0.15–41.3] years), respectively (*p* = 0.068). Of the treated patients, 47 (84%) had the neuronopathic phenotype. Of the untreated patients, 34 (71%) had this phenotype (*p* = NS).

Kaplan–Meier analysis of data from the 604 echocardiograms indicated that moderate‐to‐severe valve disease (grade 2 or higher) occurred at a significantly higher age in treated (*n* = 56) than in untreated (*n* = 48) patients (*p* < 0.0001), with ERT delaying onset by 11.5 years in 50% of patients (Figure 2A; see legend for patient characteristics). With regard to severe valve disease, time event‐free was significantly longer in patients treated with ERT than in untreated patients (Figure 2B) and median age ± SE event‐free was also significantly greater (treated, 21.1 ± 0.75 [95% CLs:19.4–23] years; untreated, 15.08 ± 0.76 [95% CLs:13.6–16.6]; Mantel–Cox regression model *χ* ^2^, 6.4 [*p* = 0.01]).

Analysis of data from the non‐neuronopathic patients only indicated that almost all patients in both the treated and untreated groups remained event‐free and did not develop severe valve disease over the course of the study (Figure 2C). The overall difference observed between the treated and untreated groups was therefore not due to the non‐neuronopathic patients. Formal analysis of the neuronopathic patients only was not possible due to the low number of data points available.

### 3.5 Cox regression modeling of long‐term ERT and valve disease

A Cox regression model adjusted for age at ERT start indicated that long‐term ERT significantly delayed the development of severe valve disease; the difference between treated and untreated patients became significant after 5 years of ERT (Figure 2D; *χ* ^2^, 32.736, *p* < 0.0001, Mantel–Cox Omnibus test for coefficients).

#### 3.5.1 Subanalysis of patients receiving ERT for more than 5 years

Forty‐three treated and 18 untreated patients had at least three follow‐up echocardiographic examinations and a time difference between first and last examination of more than 5 years. Of these 43 treated patients, 21 (49%) developed no valve disease or their grading class remained unchanged. Valve disease improved by one valve disease grading class in 12 patients (28%) and worsened in eight patients (19%; two of these patients received valve replacement). Of the untreated patients, nine (50%) showed no deterioration in valve disease, and valve disease developed or worsened in the remaining nine patients (50%). Only one untreated patient did not develop valve disease; this patient had a non‐neuronopathic phenotype and a total follow‐up of 11 years. Overall, these data indicate a 50% probability of worsening of valve status within 5 years in untreated patients, compared with an 18% probability in patients treated for more than 5 years.

## 4 DISCUSSION

This is the longest and largest single‐center, long‐term longitudinal study of the natural history of valve disease and the impact of ERT in patients with MPS II. Our data suggest that, if untreated, almost all patients will develop severe valve disease over time, highlighting the burden of cardiac disease in patients with MPS II. We show for the first time that long‐term treatment with ERT delays the onset of moderate‐to‐severe valve disease, with the difference becoming significant after 5 years of treatment. Severity of valve disease correlated with estimated GAG burden, which in turn was reduced by ERT; the degree of LVH reflected the severity of the valve disease and was not correlated with GAG burden.

Our data showing an association between estimated GAG burden and valve disease severity, and the impact of long‐term ERT on these parameters, are consistent with the hypothesis that DS has a role in the extracellular matrix of the heart and heart valves.^14^, ^15^ It is likely that the pro‐atherosclerotic, immunological and inflammatory (e.g., CD68+, CD36), pro‐apoptotic (activation of matrix metalloproteinases [MMPs] such as MMP‐9), and proliferative properties of DS and other GAGs lead to a disruption of collagen fibers, resulting in a loss of shape and function of the heart valves.^24^, ^25^ Consequently, in MPS II and other DS storage diseases such as MPS I and MPS VI, patients often develop fibrosis of the heart valves, which manifests according to the levels of accumulated GAG burden and typically in young adulthood.^26^ Patients develop rapid onset, progressive cardiac involvement, mainly a thickening of the valvular leaflets, which over time deteriorates to severe valve disease.^27^, ^28^ Consistent with previous reports,^9^, ^10^ valve involvement in this study was primarily left‐side (i.e., mitral and aortic); tricuspid and pulmonary valve involvement was rare.

Biological valves can be affected by GAG accumulation,^29^ and valve reconstruction or valve replacement with biological prostheses in patients with an MPS has a very short half‐life before the patient needs a revision, sometimes less than 1 year (personal experience of the authors). Therefore, mechanical valves should be considered whenever valve replacement is indicated. However, careful management is needed in the context of life‐long anti‐coagulation and the high number of surgical procedures needed for other MPS manifestations, for example spinal cord decompression and hip replacement. Given the high peri‐operative mortality in patients with an MPS, there is a strong need to reduce the number of potentially life‐threatening surgical interventions, including valve replacements.^9^, ^10^

We demonstrated a strong treatment effect of ERT with idursulfase, showing a significant delay in the onset of moderate‐to‐severe valve disease. The treatment effect became significant after 5 years of ERT, with these data supported by a sub‐analysis of patients who had received ERT for more than this period. Notably, the few treated patients who developed severe valve disease had all started ERT relatively late in life. The proportion of patients with the neuronopathic or non‐neuronopathic phenotype was similar in the treated and untreated groups, and similar proportion of treated and untreated non‐neuronopathic patients progressed to severe valve disease. Together, these data indicate that the treatment effect on delay in onset of severe valve disease was driven by the neuronopathic group, although the number of data points was too low to analyze this directly.

It has previously been shown that ERT stabilizes or improves LVMI.^11^, ^16^, ^17^, ^18^ Our data indicate that LVH was associated with valve disease severity, which in turn correlated with estimated lifetime GAG burden. The GAG burden was reduced by ERT, providing a mechanistic explanation for the stabilization or improvement of LVMI with ERT.

Although two recent studies did not find any impact of ERT on valve disease,^11^ there are several reasons why this may have been the case. One of these studies was able to include only a small number of patients with a limited number of cardiac assessments. The cohort in the UK study contained a greater number of patients. However, there was a high degree of LVH and cardiovascular involvement at baseline, the nature of the analysis enabled only top‐line assessment of progression of valve disease or otherwise rather than examination of the rate of progression over time, and baseline cardiac examinations were not available for all patients. A strength of our study was that echocardiograms were analyzed by a single cardiologist and that comparisons were made between treated and untreated patients. Formal grading of valve disease from a large number of echocardiograms enabled us to perform an in‐depth analysis that revealed slowing of progression of valve disease over time that would not be visible in a more top‐line analysis. Nonetheless, and as with any similar study, our analysis has some limitations. Owing to the large number of echocardiograms and the need to reduce variability by having a single cardiologist analyze the data, the severity of valve disease was assessed in grades, limiting the level of detail in descriptions of valve disease, such as whether one or more valves were affected. The study spanned a period of over 20 years from before availability of intravenous ERT until after its approval and use as standard of care, and this may have influenced the follow‐up and care that patients received. Furthermore, although the maximum duration of follow‐up for natural history and treated patients was large (>14 years), the mean follow‐up times were shorter and patient numbers were small for some timepoints used in analyses. This is likely to be because some patients were very young at the time of the analysis and had only recently initiated treatment. The analyses of lifetime GAG burden can only be considered as estimates, and it is important to consider that interpretation of differences between groups may be confounded by the natural decline observed in uGAG levels over time as children age (in both healthy children and those with MPS II).^30^, ^31^ The retrospective, observational nature of the study also has inherent limitations, and, given the challenges associated with collecting these data in a rare disease population over a long time period, statistical power was not explored. Although we were able to include a natural history group, some patients transitioned from this group to the treated group when they were able to start treatment. However, we note that age at first examination was similar in the treated and untreated groups, and that the number of patients and maximum duration of follow‐up were large for a study in MPS II.

Overall, our data provide valuable insights into the natural history of valve disease in MPS II and demonstrate for the first time that long‐term ERT delays the onset of moderate‐to‐severe valve disease. Cardiac involvement is a significant contributor to morbidity and mortality in MPS II, and mitigation of both this involvement and of the corresponding valve replacement surgeries is an important consideration in the care and long‐term outcomes for patients with MPS II. Of note, although ERT does delay the progression of valve disease it is not possible to reverse severe valvular involvement. This, coupled with the observed benefit of long‐term treatment of at least 5 years, highlights the importance of starting ERT early enough in the progression of the disease.

## AUTHOR CONTRIBUTIONS

Christoph Kampmann, Christina Lampe, Christiane M. Wiethoff, Tariq Abu‐Tair, Laila Arash‐Kaps, Eugen Mengel, Joerg Reinke, Michael Beck, and Julia B. Hennermann all made substantial contributions to the conception of the work and the analysis and interpretation of data. All authors contributed to the drafting and revision of the manuscript and have read and approved the final version for submission. All authors agree to be accountable for their contributions.

## FUNDING INFORMATION

Funding for this research study was granted by Takeda Pharmaceutical Company Limited, Tokyo, JP. The authors confirm independence from the sponsors; the content of the article has not been influenced by the sponsors. Medical writing support for this publication was funded by University Medicine Mainz, Germany, and provided by Dr. Helen Bremner, Oxford PharmaGenesis Ltd., UK.

## CONFLICT OF INTEREST STATEMENT

Christoph Kampmann, Christiane M. Wiethoff, Laila Arash‐Kaps, Joerg Reinke, and Tariq Abu‐Tair declare that they have no conflict of interest. Michael Beck has sadly now passed away, but had no conflict of interest to declare at the time of manuscript development. Christina Lampe has received travel support, speaker fees/honoraria for participating in advisory boards, research funds and consulting fees from Amicus, Alexion, Chiesi, BioMarin, Sanofi Genzyme, Takeda, Regenxbio, and Ultragenyx. Eugen Mengel has received research grants and consultation fees from Takeda, Sanofi Genzyme, Amicus, Alexion, and Orphazyme. Julia B. Hennermann has received consulting fees from Chiesi, Amicus, and Genzyme/Sanofi.

## ETHICS STATEMENT

The responsible Ethics Committee (“Ethik‐Kommission bei der Landesärztekammer” at University Medicine Mainz) issued a waiver for ethical consultation for this retrospective data analysis.

## INFORMED CONSENT

The Institutional Review Board (“Ethik‐Kommission bei der Landesärztekammer” at University Medicine Mainz) waived consent owing to the retrospective nature of the study.

## Supporting information


Figure A.

Data S1.

## Data Availability

The datasets generated or analyzed during the current study are available from the corresponding author on reasonable request.
