# Supplementary figures and images for: Natural history of valve disease in patients with mucopolysaccharidosis II and the impact of enzyme replacement therapy
Source: J Inherit Metab Dis. 2024 Oct 23;48(1):e12808. doi: 10.1002/jimd.12808 (PMC11670151; doi:10.1002/jimd.12808)

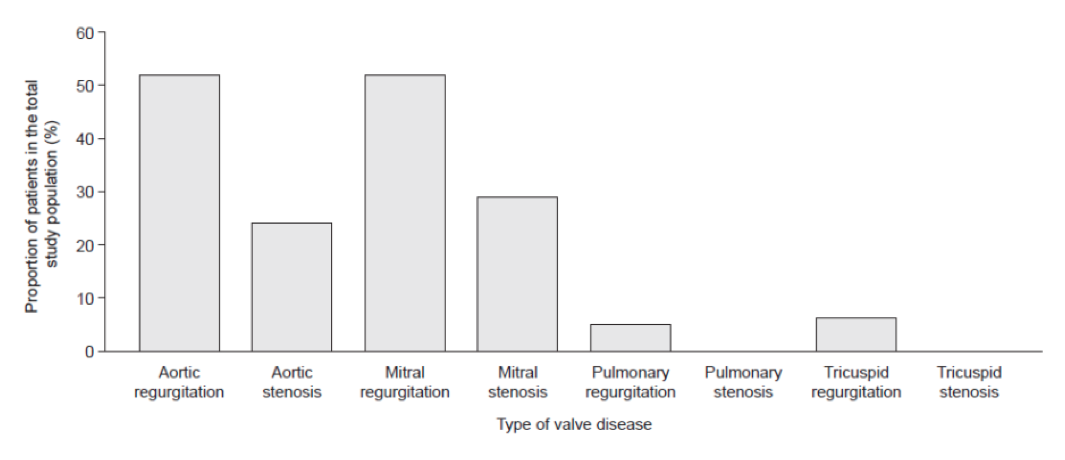

Supplement: Supplementary file 1 — Figure A. [file JIMD-48-0-s002.png]
