# Supplementary material for: Natural history of valve disease in patients with mucopolysaccharidosis II and the impact of enzyme replacement therapy
Source: J Inherit Metab Dis. 2024 Oct 23;48(1):e12808. doi: 10.1002/jimd.12808 (PMC11670151; doi:10.1002/jimd.12808)
Supplement: Supplementary file 2 — Data S1. [file JIMD-48-0-s001.docx]

**SUPPLEMENTARY INFORMATION**

**SUPPLEMENTARY TABLE A** Relationships between lifetime GAG burden, left ventricular mass and valve disease

|  | **LVH** | **No LVH** | ***p* value** |
| --- | --- | --- | --- |
| Number of patients, *N* | 23 | 55 |  |
| Lifetime GAG burden ([μg/mg_Crea_/d]*D) | 388,091 ± 97,517  422,513  (238,776–532,363) | 356,475 ± 328,952  250,000  (274,498–1,660,000) | 0.803 |
|  | | | |
|  | **Valve disease** | **No valve disease** | ***p* value** |
| Number of patients, *N* | 40 | 39 |  |
| LVMI (g/m^2^) | 109 ± 41  92.8  (53–244) | 85 ± 26  81.2  (45–185) | 0.005 |
| Lifetime GAG burden, ([μg/mg_Crea_/d]*D) | 565,485 ± 359,517  456,826  (187,758–1,660,000) | 199,831 ± 92,977  194,699  (27,498–384,251) | 0.0001 |

*Note*: data are presented as mean ± standard deviation; median; range unless otherwise stated. *p* values were generated using an independent Student’s *t*-test. There were 201 GAG measurements that could be correlated with the corresponding echocardiograms. One patient in this study did not have echocardiogram data available.

Abbreviations: Crea, creatinine; d, day; D, lifetime days; GAG, glycosaminoglycan; LVH, left ventricular hypertrophy; LVMI, left ventricular mass index.

**SUPPLEMENTARY TABLE B** Severity of valve disease in relation to MPS II phenotype and lifetime GAG burden

|  | Neuronopathic (*n*=49) | | Non-neuronopathic (*n*=31) | | *p* value |
| --- | --- | --- | --- | --- | --- |
|  | No valve disease (n=29) | Valve disease (n=20) | No valve disease (n=10) | Valve disease (n=21) |  |
| Number of patients with GAG measurements available, *n* (%) | 28 (96.6) | 8 (40.0%) | 5 (50.0%) | 18 (85.7%) |  |
| Lifetime GAG burden ([μg/mg_Crea_/d]*D) | 209,194 ± 94,149 | 537,950 ± 39,6513 | 145,518 ± 70,335 | 577,722 ± 353,303 | <0.01^*a^ |

Abbreviations: Crea, creatinine; d, day; D, lifetime days; GAG, glycosaminoglycan; MPS II, mucopolysaccharidosis II.

^*a^Significant difference in lifetime GAG burden between patients with no valve disease and those with moderate to severe valve disease in each group. There was no significant difference in lifetime GAG burden between neuronopathic and non-neuronopathic patients without valve disease, or between neuronopathic and non-neuronopathic patients with moderate-to-severe valve disease.

**SUPPLEMENTARY FIGURE A** Relative frequency of each type of valve disease in the 80 patients with MPS II in this study

AR, aortic regurgitation; AS, aortic stenosis; MR, mitral regurgitation, MS, mitral stenosis; PR, pulmonary regurgitation; PS, pulmonary stenosis; TR, tricuspid regurgitation; TS, tricuspid stenosis.
